# Drug resistance in ovarian cancer: from mechanism to clinical trial

**DOI:** 10.1186/s12943-024-01967-3

**Published:** 2024-03-28

**Authors:** Ling Wang, Xin Wang, Xueping Zhu, Lin Zhong, Qingxiu Jiang, Ya Wang, Qin Tang, Qiaoling Li, Cong Zhang, Haixia Wang, Dongling Zou

**Affiliations:** 1 Department of Gynecologic Oncology, Chongqing University Cancer Hospital & Chongqing Cancer Institute & Chongqing Cancer Hospital, Chongqing, China; 2 Chongqing Specialized Medical Research Center of Ovarian Cancer, Chongqing, China; 3 Organoid Transformational Research Center, Chongqing Key Laboratory of Translational Research for Cancer Metastasis and Individualized Treatment, Chongqing University Cancer Hospital, Chongqing, China; 4 Biological and Pharmaceutical Engineering, School of Medicine, Chongqing University, Chongqing, China

**Keywords:** Ovarian cancer, miRNAs, Resistance mechanisms, Clinical trials

## Abstract

Ovarian cancer is the leading cause of gynecological cancer-related death. Drug resistance is the bottleneck in ovarian cancer treatment. The increasing use of novel drugs in clinical practice poses challenges for the treatment of drug-resistant ovarian cancer. Continuing to classify drug resistance according to drug type without understanding the underlying mechanisms is unsuitable for current clinical practice. We reviewed the literature regarding various drug resistance mechanisms in ovarian cancer and found that the main resistance mechanisms are as follows: abnormalities in transmembrane transport, alterations in DNA damage repair, dysregulation of cancer-associated signaling pathways, and epigenetic modifications. DNA methylation, histone modifications and noncoding RNA activity, three key classes of epigenetic modifications, constitute pivotal mechanisms of drug resistance. One drug can have multiple resistance mechanisms. Moreover, common chemotherapies and targeted drugs may have cross (overlapping) resistance mechanisms. MicroRNAs (miRNAs) can interfere with and thus regulate the abovementioned pathways. A subclass of miRNAs, “epi-miRNAs”, can modulate epigenetic regulators to impact therapeutic responses. Thus, we also reviewed the regulatory influence of miRNAs on resistance mechanisms. Moreover, we summarized recent phase I/II clinical trials of novel drugs for ovarian cancer based on the abovementioned resistance mechanisms. A multitude of new therapies are under evaluation, and the preliminary results are encouraging. This review provides new insight into the classification of drug resistance mechanisms in ovarian cancer and may facilitate in the successful treatment of resistant ovarian cancer.

**Supplementary Information:**

The online version contains supplementary material available at 10.1186/s12943-024-01967-3.

## Introduction

Ovarian cancer (OC) is the third most common and the most lethal malignancy of the female reproductive system. Seventy percent of patients are diagnosed at an advanced stage (FIGO stage III and IV) with distant metastasis [1]. Despite receiving standard-of-care therapy (optimal cytoreductive surgery followed by adjuvant chemotherapy), most patients develop recurrent disease, which is resistant to chemotherapy, resulting in a 5-year survival rate of approximately 30–40% worldwide [2]. Although maintenance therapy with poly (adenosine diphosphate-ribose) polymerase (PARP) inhibitors (PARPis) has prolonged progression-free survival (PFS) and 5-year overall survival (OS) [3–5], unfortunately, many patients do not respond to PARPi treatment due to intrinsic or acquired resistance. Drug resistance is a formidable challenge in the treatment of ovarian cancer and is the primary contributor to poor prognosis.

According to the National Comprehensive Cancer Network (NCCN) guidelines (version 1.2023), there are many therapeutic regimens for resistant ovarian cancer, including some novel agents. However, the objective remission rate is still low, and the median survival time is less than 12 months due to complicated resistance mechanisms. In resistant ovarian cancer, the classical mechanisms of action of common drugs can be disrupted or altered, possibly resulting in impaired therapeutic effects. Thus, treatment regimens should not rely only on empirical options. In addition to traditional drugs, novel compounds are being investigated and tested in early clinical trials [6]. As the number of categories of agents increases, after the development of multidrug resistance (MDR), the decision of appropriate later-line therapeutic regimens is very challenging. This issue prompted us to consider the interactions of drug resistance mechanisms among different agents.

**Fig. 1:**
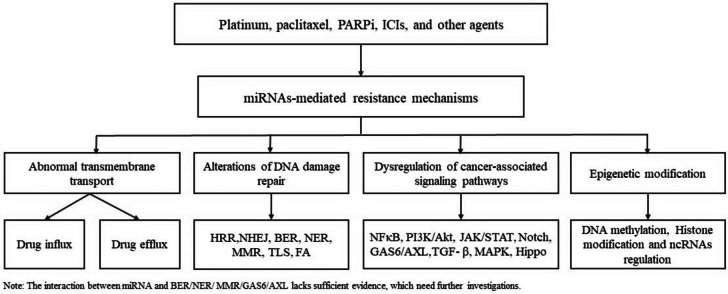
The summery of miRNA-mediated resistance mechanisms (**a**) Abnormal transmembrane transport; (**b**) Alterations of DNA damage repair; (**c**) Dysregulation of cancer-associated signal pathway; (**d**) Epigenetic modification

Even if resistance can develop to different drugs, the underlying mechanisms may be similar. Thus, instead of simply distinguishing resistance by agent, we attempted to classify drug resistance by mechanism. We summarized four major mechanisms (Fig. 1) from the published literature: 1) abnormalities in transmembrane transport, 2) alterations in DNA damage repair (DDR), 3) dysregulation of cancer-associated signaling pathways, and 4) epigenetic modifications. MicroRNAs (miRNAs) post-transcriptionally regulate the expression of target genes and affect a variety of biological processes, including cancer cell proliferation, metastasis, and therapeutic resistance [7]. miRNAs significantly regulate drug resistance by acting on molecules or/and pathways related to the four abovementioned mechanisms. Abnormal miRNA expression can lead to dysregulation of drug transporters, which control drug influx and efflux [8, 9]. The expression of some components of DDR mechanisms, such as homologous recombination repair (HRR) and nonhomologous end joining (NHEJ), is modulated by miRNAs [10]. In addition, miRNAs can interfere with multiple cancer-associated signaling pathways by targeting their components, thereby promoting tumor resistance to therapy [11].

Based on the abovementioned findings, we retrieved phase I/II clinical trials (Table 1, Figure S1 and S2) of novel drugs for resistant ovarian cancer. Understanding the underlying resistance mechanisms is expected to contribute to the identification of new clinical options for reversing resistance and improving the prognosis of ovarian cancer patients.

**Table 1:** The summary of phase I/II clinical trials about ovarian cancer with resistance based on mechanisms. Some novel agents in I/II-phase clinical trials attempt to reverse resistance in ovarian cancer by targeting transmembrane transport, DNA damage repair, signal pathway, and epigenetic modification. We summarized these components of clinical trials including the study identifier, phase, experiment drug, involved target, disease condition, primary outcome measures, study status, study results

**Study Identifier**	**Phase**	**Experiment Drug**	**Involved target **	**Disease condition**	**Primary Outcome Measures**	**Study Status**	**Study Results**
**Clinical trials targeting transmembrane transport**							
NCT04918186	II	BA3011（BA3021） + Durvalumab	Axl （ROR2）	platinum resistant high grade serous ovarian cancer	ORR	Recruiting	NA
NCT01335958	I	DMUC5754A	MUC16	Platinum-Resistant Ovarian Cancer	DLTs	Completed, published	Grade ≥3-related AE in ≥5%， anti-tumor activity in MUC16-high patients
NCT02146313	I	DMUC4064A	MUC16	Platinum-Resistant Ovarian Cancer	DLTs, MTD, PR2D, AEs, sAEs	Completed published	grade ≥ 3 toxicities in 25% RP2D: 5.2 mg/kg, CBR: 46% in MUC16-high patients
NCT01469793	I	DMOT4039A	Mesothelin	Platinum-Resistant Ovarian Cancer	MTD, DLTs RP2D	Completed， published	RP2D： 2.4 mg/kg (q3w) and 1.0 mg/kg (q1w)； SAE:8.5%
NCT02751918	Ib	BAY94-9343+PLD	Mesothelin	Mesothelin-expressing Platinum-resistant Recurrent Ovarian, Fallopian Tube or Primary Peritoneal Cancer	MTD, AEs	Completed published	ORR: 27.7% (all) ORR: 42.1% (high mesothelin expression) MTD: 6.5 mg/kg The most common AE: nausea (47.7%)
NCT01363947	I	DNIB0600A	NaPi2b	Non-mucinous and platinum-resistant ovarian cancer	AEs, DLTs, RP2D, OR, DOR	Completed published	grade ≥3 neutropenia (10%) RP2D: 2.4 mg/kg(q3w） All RECIST responses (NaPi2b-high)
NCT04504916	II	Zilovertamab Vedotin	ROR1	platinum-resistant ovarian cancer	ORR, TTR, DOR, PFS, OS	Completed	NA
NCT02539719	1a/1b	Tamrintamab pamozirine	DPEP3	platinum-resistant/refractory ovarian cancer	AEs, ORR	Completed published	ORR:4% (intolerable) Higher response in higher DPEP3
**Clinical trials targeting DDR**							
NCT02595892	II	Gemcitabine Hydrochloride + M6620	ATR	recurrent, platinum-resistant high-grade serous ovarian cancer	PFS, ORR	Completed published	Show some benefits of adding M6620 to gemcitabine (PFS: 22.9w vs 14.7w)
NCT04149145	I	M4344+Niraparib	ATR	PARPi-resistant advanced epithelial serous ovarian cancer, primary peritoneal cancer, or fallopian tube cancer	AEs, MTD	Not yet recruiting	NA
NCT03462342	I	AZD6738+ Olaparib	ATR	Recurrent platinum-sensitive and platinum-resistant HGSOC	AEs, ORR, PFS	Recruiting	NA
NCT03704467	Ib//II	Carboplatin + M6620 + Avelumab	ATR	PARPi-resistant Ovarian Cancer	DLT, AEs	Recruitment completed	NA
NCT01164995	II	MK-1775 + carboplatin	WEE1	Refractory or Platinum Resistant Ovarian Cancer with TP53 mutation	AEs, antitumor activity (CT/CA125)	Completed published	ORR: 41%, PFS: 5.6m AE: bone marrow toxicity, nausea and vomiting
NCT03579316	II	AZD1775+ olaparib	WEE1	PARPi-resistant ovarian cancer	ORR, safety and tolerability	Recruiting	NA
NCT02272790	II	Adavosertib+ Carboplatin/ PLD/Paclitaxel/ Gemcitabine	WEE1	Platinum-Resistant Epithelial Ovarian, Fallopian Tube, or Primary Peritoneal Cancer	ORR, AEs	Completed published	ORR (overall):31.9% ORR (adavosertib+ Carboplatin):66.7% Grade ≥3 AEs: anemia (33%), neutropenia (45.7%), Thrombocytopenia (31.9%)
NCT05198804	I/II	ZN-c3 + Niraparib	WEE1	Platinum-/PARPi-Resistant Ovarian Cancer	DLTs, PFS, ORR	Recruiting	NA
NCT04516447	I	ZN-c3+ PLD/carboplatin/ paclitaxel/gemcitabine	WEE1	Platinum-Resistant Ovarian, Peritoneal or Fallopian Tube Cancer	AEs, MTD, RP2D	Recruiting	NA
NCT02101775	II	Gemcitabine with or without MK-1775	WEE1	Recurrent, Platinum Resistant Epithelial Ovarian, Primary Peritoneal, or Fallopian Tube Cancers	PFS, OR, OS, AEs	Completed published	PFS: 4.6 months vs 3.0 months (HR 0.56, 95%CI:0.35 to 0.90, *p*=0.015). OS: 11.5 months vs 7.2 months (HR 0.56, 95%CI 0.34 to 0.92, *p*=0.022). PR rate: 21% vs 3% (*p*=0.02)
NCT02203513	II	LY2606368	CHK1/2	recurrent platinum-resistant HGSOC with BRCA wild-type or mutation	ORR	Partially completed (BRCA wide-type), recruitment ongoing	PR (assessable per protocol): 33% (8/24), Grade≥3AEs: neutropenia (93%); reduced white blood cell count (82%); thrombocytopenia (25%), anemia (11%).
NCT03414047	II	LY2606368	CHK1/2	platinum-resistant HGSOC with BRCA wild-type or mutation	ORR	Completed	In platinum resistant patients: ORR (Cohorts 1--3): 12.1% DCR was 37.1%,
NCT04678102	I	PHI-101	CHK2	Platinum Resistant Ovarian, Primary Peritoneal, or Fallopian Tube Cancers	DLT, MTD	Recruiting	NA
NCT02797964	I/II	SRA737	CHK1	Platinum-resistant or intolerant HGSOC	AEs, MTD	Completed	MTD: 1000 mg QD RP2D: 800 mg QD Mild toxicities
**Clinical trials targeting signaling pathway**							
NCT03875820	I	Defatcinib+VS-6766	MAPK	LGSOC without conventional treatment	Estabilsh tolerated dose and Measure. AEs	Active, not recruiting	NA
NCT03648489	II	TAK228	PI3K/AKT/mTOR	platinum-resistant ovarian cancer	PFS	Active, not recruiting	NA
NCT03586661	I	Copanlisib	PI3K/AKT	platinum-resistant ovarian cancer with BRCA mutation	MTD and RP2D	Active, not recruiting	NA
NCT04374630	II	Afuresertib+paclitaxel	PI3K/AKT	Platinum-Resistant Ovarian Cancer	PFS	Recruiting	NA
NCT04586335	I	CYH33	PI3K/AKT	Platinum-/PARPi-Resistant Ovarian Cancer	DLT, ORR	Recruiting	NA
NCT04055649	II	ONC201	PI3K/AKT、MAPK	Platinum Refractory or Resistant Ovarian Cancer	AEs, DLT's, ORR, PFS	Recruiting	NA
NCT05295589	II	Copanlisib	PI3K/AKT	Recurrent Platinum Resistant Ovarian Cancer	PFS	Not yet recruiting	NA
NCT03363867	II	Cobimetinib	MAPK	Recurrent Platinum Resistant High Grade Serous Ovarian Cancer	ORR	Recruiting	NA
NCT03639246	I/II	AVB-S6-500	GAS6-AXL	platinum-resistant Recurrent Ovarian Cancer	AEs PFS	Completed published	ORR (AVB-500 + PAC): 34.8% median DoR, PFS, and OS (AVB-500 + PAC)：7.0, 3.1, and 10.3 months, respectively RP2D (AVB-500): 15 mg/kg
NCT04019288	I/II	AVB-S6-500	GAS6-AXL	Platinum-Resistant Ovarian cancer	AEs	Completed published	no DLTs and grade ≥3 AEs within 6-week Exploratory studies are ongoing.
NCT04893551	I	Tilvestamab	GAS6-AXL	Platinum-resistant relapsed HGSOC	AEs	Terminated	NA
NCT01952249	Ib	Demcizumab+paclitaxel	Notch	platinum-resistant ovarian, primary peritoneal, and fallopian tube cancer	DLTs	Completed	RP2D: 3.5mg/kg tolerability, clinical activity,
NCT03776812	II	Relacorilant + Nab-Paclitaxel	GR	recurrent platinum-resistant ovarian, fallopian tube, or primary peritoneal cancer	PFS, ORR, DOR	Completed	ORR is similar among arms (35%); Intermittent arm: OS, 13.9 months, PFS, 5.6 months Continuous arm; OS months, 11.3, PFS, 5.3 months Nab-paclitaxel: OS, 12.2 months, PFS, 3.8 months
NCT03319628	1/II	XMT-1536	NaPi2b	platinum-resistant ovarian cancer	MTD and RP2D	Recruiting	NA
NCT04502602	1/1b	Niraparib + Neratinib	HER2	Platinum-resistant Ovarian Cancer	RP2D, PFS, AEs	Recruiting	NA
NCT03287271	I/II	Defactinib (VS-6063) +Carboplatin/PaclitaxeL	FAK	Carboplatin-resistant Ovarian Cancer	ORR, AEs	Recruiting	NA
**Clinical trials targeting epigenic modification**							
NCT05327010	II	ZEN003694 +Talazoparib	BET bromodomain	PARPi-resistant recurrent ovarian cancer with BRCA mutation or DDR aberrations	ORR	Recruiting	NA
NCT04840589	I	ZEN003694+nivolumab+/- Ipilimumab	BET bromodomain	Recurrent Platinum-Resistant Ovarian Carcinoma	RP2D	Recruiting	NA
NCT03206047	I/II	atezolizumab+/-Guadecitabine+/-CDX-1401 vaccine	DNMT	platinum-resistant epithelial ovarian, fallopian tube, or primary peritoneal carcinoma	AEs, PFS	Recruiting	NA
NCT02901899	II	Guadecitabine + Pembrolizumab	DNMT	recurrent platinum resistant ovarian cancer	ORR	Complete	PR: 8.6%; SD:22.9%; CBR: 31.4% (95% CI: 16.9%-49.3%); duration of clinical benefit was 6.8 months

*ORR* Objective Response Rate, *DLTs* Dose-Limiting Toxicities, *MTD* Maximal Tolerance Dose, *PR2D* Recommended Phase II Dose, *AEs* Adverse Events, *sAEs* severe Adverse Events, *PFS* Progression-Free Survival, *CBR* Clinical Benefit Rate, *GR* the glucocorticoid receptor, *DoR* Duration of Response

## Mechanisms of drug resistance in ovarian cancer

### Abnormal transmembrane transport

**Fig. 2:**
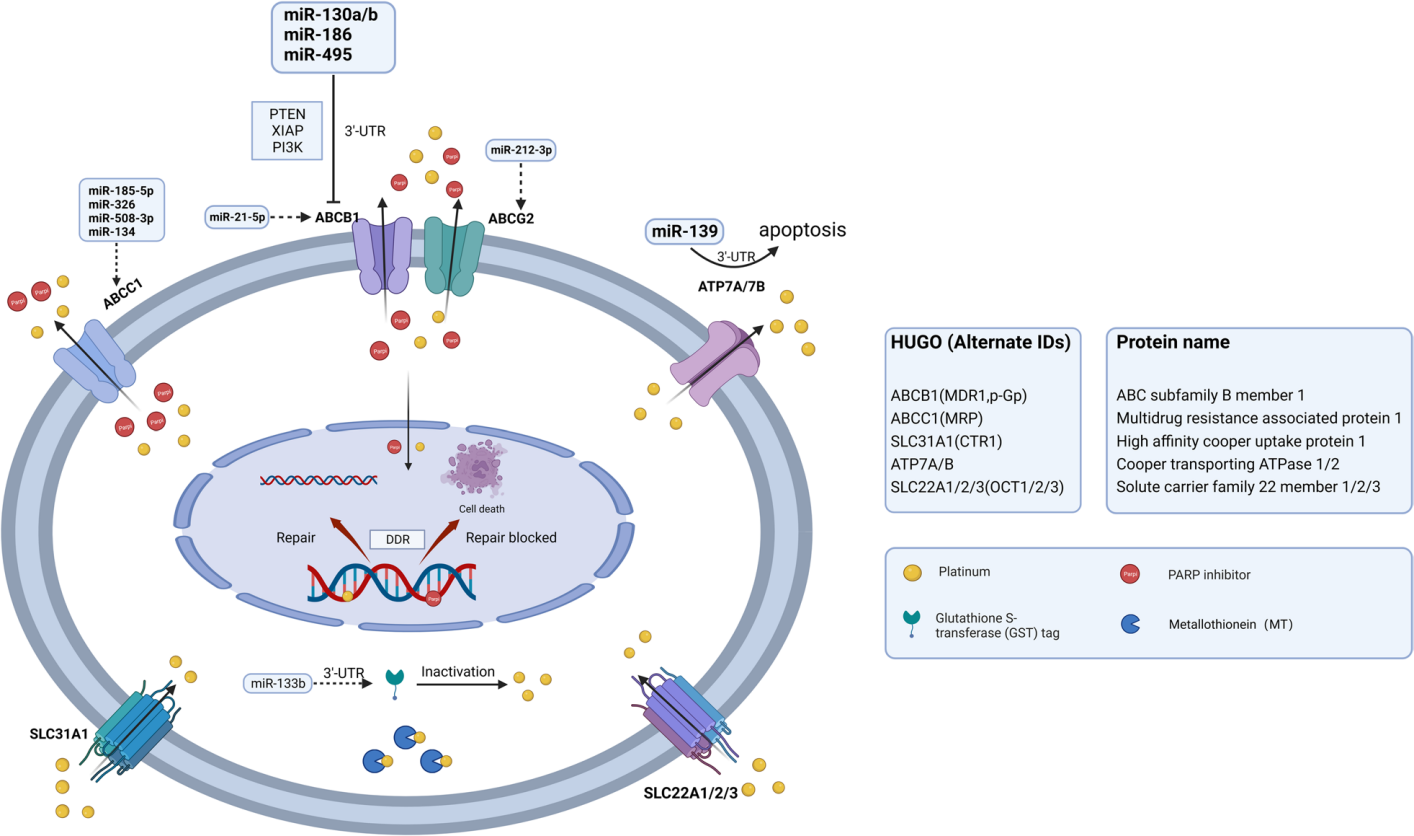
Abnormal transmembrane transport. The SLC31A1, SLC22A1/2/3, as members of SLC superfamily, are significant transporters in charge of drug inflow. Downregulation of SLC transporters reduce platinum uptake, leading to chemoresistance in ovarian cancer. The role of miRNA in SLC expression lacks sufficient evidence. The ABC transporter family include ABCB1, ABCG2, ABCC1, which are responsible for drug efflux and then reduce intracellular concentration of platinum. miR130a/b, miR-186, miR-495 can directly bind with the 3'-UTR of ABCB1 mRNA or regulate PTEN, XIPA, and PI3K, leading to decreased ABCB1 transcription or translation level. miR-21-5p and miR-212-3p also have a regulatory factor of ABCB1 and ABCG2, respectively. miR-185-5p, miR-326, miR-508-3p and miR-134 can regulate the expression of ABCC1. ATP7A/7B are another contributor of drug efflux. miR-139 can directly bind to the 3'-UTR of ATP7A/7B, leading to apoptosis induction and increasing the chemosensitivity of ovarian cancer. MT can bind to cisplatin and deactivates it, which decreases drug efficacy and induces drug resistance. GST catalyzes glutathione to bind platinum and causes drug inactivation, which is associated with platinum resistance in ovarian cancer. (SLC, solute carrier superfamily; GST, Glutathione transferase; MT, Metallothionein)

Decreased influx and increased efflux are two forms of abnormal transmembrane transport that reduce the intracellular drug concentration and result in resistance (Fig. 2). Moreover, in platinum-resistant ovarian cancer (PROC), the expression of the related genes and transporters is decreased. Thus, the intracellular concentration of platinum is insufficient, and platinum resistance subsequently develops [12–16]. miRNAs can directly target transmembrane transporters, thereby regulating cellular resistance to drugs [17]. They directly bind to the 3'-untranslated region (3'-UTR) of a targeted transporter gene to regulate its transcription, leading to abnormalities in drug influx and efflux [18].

#### Reduced drug influx

Sodium pumps, copper ion transporters, and organic cation transporters on the cell membrane or plasma membrane, such as the drug-transporting solute carrier (SLC) superfamily transporters (e.g., SLC31A1, SLC22A1/2/3), are key transporters controlling drug influx. SLC31A1 has been convincingly demonstrated to transport cisplatin and its analogs carboplatin and oxaliplatin, leading to intracellular accumulation of platinum [19]. The low expression of SLC22A2 in ovarian cancer may correlate with platinum drug resistance via a reduction in platinum uptake [20]. miRNAs play pivotal roles in the expression of drug-transporting SLC transporters and may influence treatment responses in prostate cancer, hepatocellular carcinoma and colorectal cancer [9]. However, the association and interaction mechanisms of miRNAs and SLC transporters in drug resistance in ovarian cancer have not been investigated, and further research is warranted.

#### Increased drug efflux

The ABC transporter family is mainly responsible for drug efflux. Abnormal expression of miRNAs (e.g., the miR-200 family, let-7 family and miR-130a/b) plays a role in ABC transporter regulation, thereby inducing resistance in ovarian cancer [21]. The characterized efflux transporters in the ABC family include ABCB1, ABCG2 and ABCCs [8, 22]. The abovementioned miRNAs can bind to the 3'-UTRs of ABC transporter-encoding mRNAs, or participate in imperfect base pairing with genes encoding nuclear receptors, transcription factors (TFs), and signaling molecules associated with ABC transporters. Through this action, the mRNAs of ABC transporters are degraded or the translation of the corresponding proteins is inhibited [8]. In addition, the vault protein lung drug resistance-related protein (LRP) can transport cytostatic drugs from intracellular targets, conferring drug resistance [23].

Whole-genome microarray analysis revealed that *ABCB1* was the only drug transporter with increased expression in resistant ovarian cancer cells, while the expression of several other ABC transporters was significantly decreased [24]. The membrane transporter P-glycoprotein (P-gp) is encoded by *ABCB1* and is an ATP-dependent drug efflux pump. Its overexpression in resistant cell lines is considered the crucial mechanism of resistance to paclitaxel, doxorubicin, sorafenib [25], and PARPis [26]. Notably, dysregulated miRNAs can mediate the overexpression of ABCB1, resulting in MDR. For instance, miR130a/b, miR-186, and miR-495 can directly bind to the 3'-UTR of ABCB1 mRNA or regulate the expression of other targets (e.g., PTEN, XIAP, and PI3K) [11, 27], leading to ABCB1 mRNA degradation or translational inhibition. A strong increase in ABCB1 expression was found to correlate with decreased expression of miR-21-5p, but the regulatory mechanism involved remains unknown [21]. In addition, upregulation of *ABCB1* is associated with the transcriptional fusion of ABCB1 and SLC25A40, which was identified through whole-genome analysis in patients with high-grade serous ovarian cancer (HGSOC) who underwent prior chemotherapy and targeted therapy [28]. These findings indicate that ABCB1 upregulation frequently induces cross-resistance to chemotherapeutics and targeted drugs. Therefore, PARPis that are not dependent on the P-gp transporter might show greater therapeutic efficacy in patients who have received chemotherapy [24]. ABCC1 is associated with poor survival and chemoresistance in HGSOC. miR-185-5p and miR-326 both target the ABCC1 3'-UTR to regulate the expression of ABCC1 [2]. miR-508-3p [29] and miR-134 [30], which are sponged by CircETDB1 and LINC01118, respectively, can posttranscriptionally regulate the expression of ABCC1. ABCG2 is involved in topotecan resistance in ovarian cancer, which is associated with miR-212-3p downregulation [31].

In addition, upregulation of the copper efflux transporters ATP7A and ATP7B contributes to chemoresistance in ovarian cancer [32]. miR-139 can directly bind to the 3'-UTR of ATP7A/B, contributing to apoptosis induction and increasing the chemosensitivity of ovarian cancer cells [33].

#### Drug inactivation

Metallothionein (MT) and glutathione (GSH) are two major thiol-containing proteins that bind to platinum-based drugs. Detoxification of cisplatin by intracellular thiol-containing proteins is considered a major hurdle to overcome. MT binding to cisplatin can induce drug resistance, which can be reversed by short hairpin MT (shMT) [34]. GSH reacts with cisplatin to form a GS-platinum complex, reducing the available intracellular platinum content [35]. Glutathione S-transferase (GST) catalyzes the binding of GSH to platinum and causes drug inactivation, which is associated with platinum resistance in ovarian cancer [36, 37].

### Alterations in DDR

**Fig. 3:**
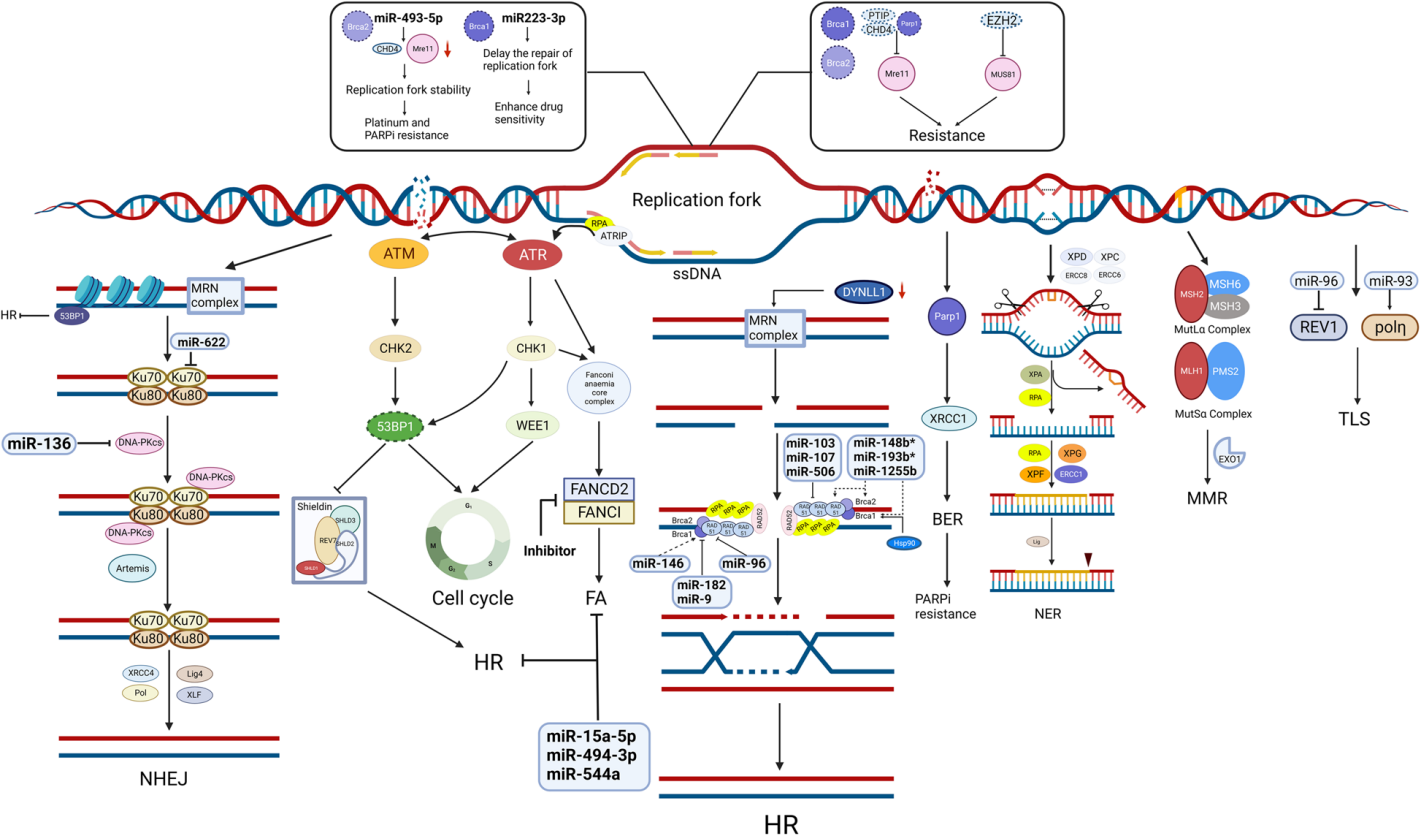
Alterations of DNA damage repair. DDR generally consists of HRR, NHEJ, Replication fork, BER, NER, MMR, TLS, and FA. The repair of DSBs occurs predominately through NHEJ repair pathway in conjunction with HRR pathway. NHEJ are initiated by binding of Ku70–Ku80 heterodimer to DNA ends. The subsequent recruitment and autophosphorylation of DNA-PKcs bring the DNA ends together and allow their ligation by XRCC4–LIG4. MRN complex (MRE11-RAD50-NBS1), an important repair factor of HRR, detects the DNA damage firstly and activates downstream signaling. Besides, it exerts nuclease activity to resect DNA end, guiding to HRR. Further, DYNLL1 binds directly to MRE11 to limit its end-resection activity. Decreased DYNLL1 restores HR-mediated double-strand DNA breaks repair. Replication fork protection is a modality independent of DSBs, which contributes to gene stabilization, leading to chemoresistance and PARPi resistance. Additionally, down-expression of 53BP1 protein is another mechanism to restore DNA end resection. Shieldin (SHLD1, SHLD2, SHLD3 and REV7), as an effector complex of 53BP1, can mediate 53BP1 dependent DNA repair in a BRCA-independent manner. The kinases ATR and ATM have crucial roles in DDR pathway, such as maintaining replication fork stability and regulating CHK1 and CHK2.CHK1 can activate the G2/M inhibiting kinase WEE1 to maintain genomic integrity. Some miRNAs were shown to regulate the expression of components involved in HRR, NHEJ, Replication fork protection, TLS, and FA, but the interaction between miRNA and BER/ NER/ MMR lack sufficient evidence. (SLC, solute carrier superfamily; GST, Glutathione transferase; MT, Metallothionein)

If DNA damage is not repaired promptly, cellular senescence or apoptotic signals are activated, while abnormal activation of DDR maintains the viability of cancer cells, significantly inducing resistance to chemotherapeutic drugs and PARPis and affecting therapeutic efficacy [38]. DDR generally consists of seven pathways (Fig. 3): the HRR, NHEJ, base excision repair (BER), nucleotide excision repair (NER), mismatch repair (MMR), translesion DNA synthesis (TLS), and Fanconi anemia (FA) pathways. Interactions among the DNA damage response, DNA repair components and miRNAs have been reported [39]. The ectopic expression of miRNAs, as regulatory factors, can interfere with the activity of DNA repair mechanisms, which have been implicated in multiple types of resistance [40]. Some miRNAs can reverse drug resistance by targeting genes encoding DDR-related enzymes [41].

#### HRR

HR deficiency is characteristic of many HGSOC cases (approximately 50%) and is considered a predictive biomarker of sensitivity to platinum agents and PARPis [42]. Restoration of HR pathway activity likely results in acquired resistance to platinum agents and PARPis in ovarian cancer patients with HR deficiency [43]. Notably, miRNAs have been revealed to impede DDR by directly targeting components of the DDR response, leading to reduced drug resistance [44]. miR-146 targets BRCA1 and is associated with the response to double-strand breaks (DSBs) [45]. Overexpressed miR-182 and miR-9 mediate the downregulation of BRCA1 and increase sensitivity to cisplatin and PARPis in ovarian cancer [46, 47]. miR-96 directly targets the coding region of RAD51 and the 3'-UTR of REV1 and decreases the efficiency of HRR [43]. miR-1255b, miR-193b*, and miR-148b* (“*” indicates minor products at low concentrations) can target the transcripts of the HR-mediated DSB repair factors BRCA1, BRCA2, and RAD51, respectively, thereby regulating PARPi sensitivity [48]. miR-506, miR-103 and miR-107 are robust clinical markers for the chemotherapy response and survival in patients with ovarian cancer and can sensitize cancer cells to DNA damage by directly targeting RAD51 and inhibiting the formation of RAD51 foci [49, 50]. Importantly, reversion mutations in *BRCA1*/2, *RAD51C*, and *PALB* have been identified during prolonged exposure to platinum agents and PARPis and in post-progression biopsies. The restoration of the open reading frame by these mutations leads to the functional restoration of HRR [51, 52]. Furthermore, HSP90 was found to mediate the stabilization of BRCA1, which interacts with the PALB2-BRCA2-RAD51 complex. This interaction was found to be essential for RAD51 focus formation and for conferring PARPi and cisplatin resistance [53]. Combination therapy with an HSP90 inhibitor and platinum is an innovative antitumor strategy that has the potential to reverse platinum resistance in ovarian cancer [54, 55].

The MRE11-RAD50-NBS1 (MRN) complex, an important factor of HRR, first detects DNA damage and then activates signaling molecules [56]. In addition, it exerts nuclease activity to resect DNA ends, guiding HRR. Furthermore, recombinant human cytoplasmic dynein light chain 1 (*DYNLL1)* was found to bind directly to MRE11 to limit its end resection activity. Thus, downregulation of *DYNLL1* restores HR-mediated DNA DSB repair, thereby inducing chemoresistance and PARPi resistance in ovarian cancer [57]. Additionally, loss-of-function mutations in the *TP53BP1* gene result in decreased 53BP1 protein expression and facilitate BRCA1-independent DNA end resection, which accounts for platinum and PARPi resistance [58].

Given the expanding role of immune checkpoint inhibitors as therapeutic agents, the interaction of tumor DNA damage and repair with the immune response has recently come into focus. HGSOC patients with BRCA mutation and homologous recombination deficiency (HRD) were found to exhibit increases in CD3 + /CD8 + tumor-infiltrating lymphocytes (TILs), immunohistochemical staining for PD-1/PD-L1, and neoantigen load. Moreover, wild-type BRCA1/2 ovarian tumors with mutations in RAD51, ATM, and ATR had higher predicted neoantigen levels than HR-proficient tumors [59, 60]. Mu Chen et al. showed that DNA damage resulted in the production of many DNA fragments in the cytoplasm, leading to increased antigen presentation on the cell surface and activation of the immune response [61]. However, a clinical trial of avelumab did not show an improved response in patients with *BRCA1/2*-mutated ovarian cancer (NCT01772004). Thus, additional clinical trials are warranted to determine the complexities of the interactions between DNA damage and immunomodulatory agents.

#### NHEJ

NHEJ repairs DNA DSBs by competing with HRR during the repair process, and its machinery includes TP53BP1, DNA-PK, etc. [62] miRNAs play important roles in regulating the expression of these NHEJ-related genes [39]. miR-136 overexpression downregulates DNA-PK, cell cycle-related genes, and antiapoptotic genes, resensitizing ovarian cancer cells to paclitaxel [63]. miR-622 suppresses NHEJ and facilitates HR-mediated DSB repair by targeting the Ku complex. Therefore, high expression of miR-622 in BRCA1-deficient HGSOC cells induces platinum and PARPi resistance [64]. DNA-PK, composed of DNA-PKcs and the DNA end-binding Ku70/80 heterodimer, has emerged as an intriguing therapeutic target within the NHEJ pathway [65, 66]. This heterodimer can recognize DSBs and form the Ku-DNA complex, which can recruit DNA-PKs to DSB sites [67]. DNA-PKcs plays a major role in promoting NHEJ through autophosphorylation and recruitment of downstream effectors, such as endonucleases (Artemis) [68] and polymerases (DNA POLM (Pol µ) and POLL (Pol λ)) [69, 70]. DNA-PK inhibition was found to induce restoration of HR function and resulted in resistance to PARPis in patient-derived ovarian cancer xenografts [71]. Ectopic expression of XRCC5/Ku80 [66] and XRCC6/Ku70 [65] induces platinum and PARPi resistance. Crucially, TP53BP1 can promote NHEJ and reduce BRCA1-mediated HRR by restricting DSB resection and antagonizing BRCA2/RAD51 loading in BRCA1-deficient cells [72]. The shieldin complex (comprising SHLD1, SHLD2, and SHLD3), an effector complex of 53BP1, regulates 53BP1-dependent NHEJ in various settings and impacts resistance to PARPis in HRD-defective cells [73, 74]. Finally, XRCC4, DNA ligase IV (LIG4) and XLF are central components of end ligation.

#### Replication fork protection

Replication fork protection contributes to genome stability in a manner independent of DSB-induced HR, leading to chemoresistance and PARPi resistance [75]. PARP1, BRCA1 and BRCA2 play key roles in protecting the replication fork under replication stress (RS) conditions [76, 77]. PTIP, PARP1 and CHD4 deficiency in BRCA-deficient cells prevent the recruitment of the MRE11 nuclease to stall replication forks and subsequently protect nascent DNA from degradation, thus conferring chemoresistance and PARPi resistance [78]. In both cells and patients with BRCA2 mutation, EZH2 downregulation leads to inhibition of the MUS81 nuclease, which restores DNA replication fork protection, leading to PARPi resistance [79]. miRNA-493-5p significantly preserves replication fork stability in BRCA2-mutant ovarian cancer cells through downregulation of *MRE11* and *CHD4*, conferring platinum and PARPi resistance [10]. However, restoration of miR223-3p expression, which delays the repair of the replication fork, leads to genomic instability and enhances drug sensitivity in BRCA1-deficient OC [80].

#### NER and BER

NER is responsible for repairing single-stranded DNA damage, and 8% of HGSOC patients exhibit alterations in some NER genes, according to The Cancer Genome Atlas (TCGA) database [81]. The NER signaling pathway can repair platinum-induced adducts, therefore, upregulation of NER genes, including ERCC1, *ERCC2-XPD, ERCC3-XPB, ERCC4-XPF, ERCC5-XPG, ERCC6, ERCC8 and XPA,* might mediate chemoresistance [63]. Indeed, overexpression of ERCC1 or XPF not only increased platinum resistance but also decreased the toxicity of olaparib [82]. Although certain NER gene mutations (ERCC6-Q524* and ERCC4-A583T) were found to be functionally associated with platinum sensitivity in vitro, these NER alterations did not affect HR or confer sensitivity to PARPis.

BER is accelerated by PARPs and the scaffold protein XRCC1. Currently, it has been reported that the BER pathway has both positive and negative associations with platinum resistance. Although BER pathway intermediates underlie the efficacy of PARPis, they mediate the activity of PARP family proteins (especially PARP1) to initiate repair, resulting in PARPi resistance.

#### MMR deficiency

MMR defects in OC are relatively underinvestigated, although they are the most common cause of hereditary ovarian cancer after BRCA1/2 mutations. The MMR pathway contains seven proteins (MSH2, MSH3, MSH6, MLH1, MLH3, PMS1, and PMS2) [66]. The frequency of MMR deficiency (loss of any protein) reportedly ranges from 2 to 29% in patients with ovarian cancer [67]. A small number of studies have suggested that MMR deficiency is associated with drug resistance, but the results were inconclusive [83–86]. The possible role of MMR defects in drug resistance in ovarian cancer deserves further investigation. Currently, MMR deficiency is proposed to occur due to loss of ineffective MMR activity, replication fork stalling, the inability to recognize DNA damage, an increase in the net replicative bypass of cisplatin adducts and modulation of the level of recombination-dependent bypass [87, 88].

#### Other DDR pathways

The FA core complex consists of at least 10 FA-associated proteins (FANCA, FANCB, FANCC, FANCE, FANCF, FANCG, FANCL, FAAP100, FAAP20 and FAAP24) [89]. Inhibition of components of the FA repair pathway such as FA complementation group D2 (FANCD2) and FANCI, can increase sensitivity to chemotherapeutic agents [90]. miR-15a-5p, miR-494-3p and miR-544a potentially inhibit the entire FA/HR pathway [91].

TLS is mediated by DNA polymerases (e.g., Pol η and REV1). It increases the tolerance of tumor cells to platinum-induced DNA adducts and results in platinum resistance [92]. Pol η and REV1 are translesion DNA polymerases [93]. Upregulation of miR-93 might reverse resistance through targeting of DNA Pol η [92]. It has been reported that miR-96 can prevent the emergence of chemoresistance by inhibiting REV1-mediated TLS.

### Dysregulated cancer-associated signaling pathways

**Fig. 4:**
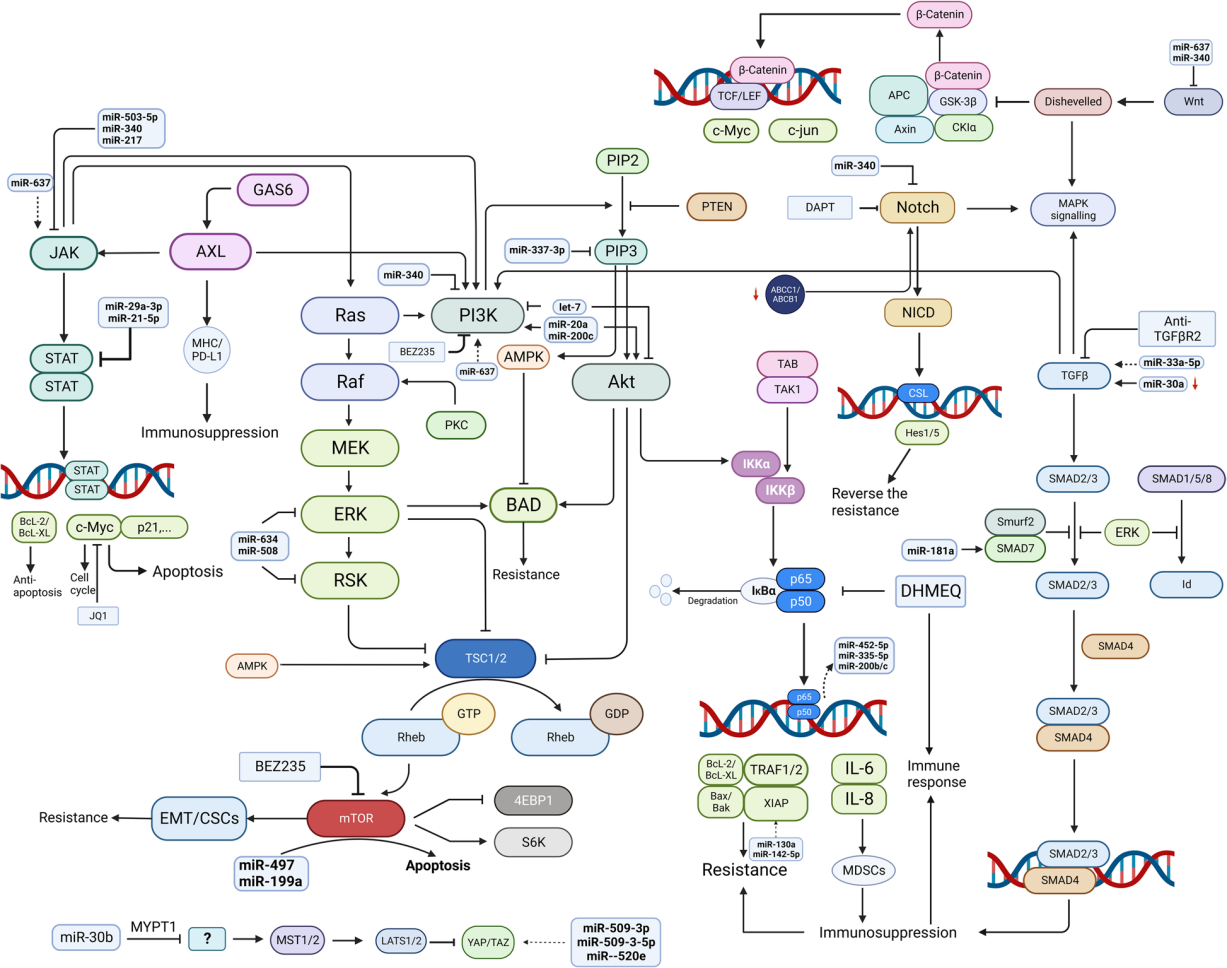
Dysregulation of cancer-associated signal pathway. A series of signal pathways collectively regulates the biological process in human malignancies, which is associated with the proliferation, invasion and therapeutic resistance. The signaling pathways mainly include NFκB, PI3K/Akt, JAK/STAT, Notch, GAS6/AXL, TGF-β, MAPK, Hippo/YAP patwhay. Some miRNAs have ability to regulate the key members of these mentioned pathway, including JAK/STAT, GAS/AXL, MAPK, PI3K/Akt, NFκB,, TGF-β, Hippo/YAP, but there are no investigations about the interaction between miRNAs and Notch in ovarian cancer. The dysregulated cancer-associated signal pathway interfere with apoptosis, cell cycle, and immune status, resulting in multidrug resistance. Molecule targets in these pathway may provide a new approach for drug resistance in OC. The γ-secretase inhibitor DAPT, c-Myc targeting small molecule JQ1, an inhibitor of NFκB DHMEQ suppress the proliferation and induce apoptosis to reversing drug resistance in OC. (JQ1, novel cell-permeable small molecule; BAD, Bcl-2 antagonist of death; IKKα, inhibitor of nuclear factor-κB subunit-α; mTOR, mammalian target of rapamycin; NF-κB, nuclear factor-κB; DHMEQ, Dehydroxymethylepoxyquinomicin; MDSCs, Myeloid-derived suppressor cells; CSCs, cancer stem cells;BEZ235,a dual PI3K/mTOR inhibitor; DAPT, γ-secretase inhibitor N-[N-(3,5-difluorophenacetyl)-L-alanyl]-S-phenylglycine t-butyl ester)

A series of signaling pathways (Fig. 4) collectively regulate biological processes in human malignancies and are associated with the proliferation, invasion and therapeutic resistance of cancer cells [94]. The expression of signaling pathway components can be modulated by miRNAs through miRNA–mRNA binding, typically to miRNA target sites in the mRNA 3’-UTR [40, 95]. Although cancer-associated signaling pathways are complex, the identification of potential therapeutic targets is promising.

#### NFκB signaling pathway

NFκB can perform a biphasic function in ovarian cancer. It plays an anticarcinoma role in ovarian cancer cells and renders them sensitive to apoptosis induced by carboplatin and paclitaxel, but it also has carcinogenic effects on promoting aggressiveness and chemoresistance in ovarian cancer cells and confers resistance to these therapeutic agents [96]. Common chemotherapeutic drugs, including taxanes, platinum agents, vinca alkaloids and erlotinib, activate NFκB and its prosurvival downstream targets, which contribute to chemoresistance [97]. Activation of the NFκB pathway is correlated with platinum resistance and leads to poor prognosis in patients with ovarian cancer [98]. Mechanistically, increased nuclear translocation of the p65 subunit and phosphorylation of inhibitor of IκB kinase subunits alpha and beta are markers of NFκB activation, which promotes chemoresistance [99]. Moreover, NF-κB p65 increases miR-200b/c expression by binding to its promoter, subsequently sensitizing ovarian cancer cells to cisplatin [100]. It also regulates the downstream miRNAs miR-452-5p and miR-335-5p through the NF-κB TFs RelA and RelB, preventing the recurrence of OC [101]. Moreover, the NF-κB signaling pathway has been implicated in immunosuppression and immune evasion in ovarian cancer cells partly via NFκB-dependent production of IL-6, which impairs DCs but generates and recruits immunosuppressive MDSCs, and IL-8, which increases the expression of the immunosuppressive enzyme arginase [102]. Dehydroxymethylepoxyquinomicin (DHMEQ), an inhibitor of NFκB, induces apoptosis, increases the response to platinum-based drugs and reverses immunosuppression in ovarian cancer cells [102, 103].

#### PI3K/Akt pathway

The PI3K/Akt pathway is frequently upregulated in ovarian cancer, and activated PI3K/Akt signaling contributes to increased cancer cell chemoresistance [104, 105]. Many miRNAs have been found to modulate the PI3K/Akt pathway, influencing ovarian cancer chemosensitivity [106]. miR-337-3p directly targets PIK3CA and PIK3CB, suppresses the proliferation of epithelial ovarian cancer cells and reverses resistance [107]. The let-7 miRNA family deregulates this pathway by governing PI3K and Akt1 phosphorylation and activity [108]. However, miR-20a and miR-200c activate and upregulate this pathway, contributing to paclitaxel resistance [109]. The aberrant PI3K-Akt signaling in tumor cells is attributed to the platinum-resistant phenotype, and the combination of cisplatin and LY-294002 (a PI3K-Akt dual kinase inhibitor) was found to prevent 3D spheroid formation and sensitize cells to cisplatin [110]. Furthermore, mTOR is a key downstream signaling kinase in the PI3K/Akt pathway [111]. Activated mTOR signaling can trigger epithelial–mesenchymal transition (EMT) and promote the maintenance of cancer stem cells (CSCs), resulting in chemoresistance in ovarian cancer patients, and treatment with BEZ235 (a dual PI3K/mTOR inhibitor) might be a promising approach for reversing chemoresistance [112]. In addition, miR-497 and miR-199a were found to quantitatively control mTOR expression to induce apoptosis in ovarian cancer cells [106].

#### JAK/STAT pathway

Following the phosphorylation of JAK, STAT is phosphorylated and activated, after which its nuclear translocation induces the transcription of its target genes involved in growth and apoptosis. M Koti et al. reported that STAT1 was the most significantly differentially expressed gene between chemoresistant and chemosensitive HGSOC. Upregulation of STAT1 is associated with platinum resistance [113]. c-Myc is a downstream target of the JAK/STAT signaling pathway and is linked with the malignancy and chemotherapeutic response of OC [114]. The novel cell-permeable small molecule JQ1 can target c-Myc to suppress the proliferation and induce the apoptosis of OC cells. Along with chemotherapeutic agents and PARPis, JQ1 warrants further investigation regarding its ability to reverse drug resistance in OC patients through interaction with the JAK-STAT signaling pathway [115]. This pathway is also regulated by miRNAs, and miRNA interactions are linked to drug resistance. Restoration of miR-503-5p expression can block the downstream JAK2/STAT3 pathway through the binding of this miRNA to the 3’-UTR of the mediator CD97 [116]. miR-340 can also directly target LGR5, FHL2, CTNNB1, and BAG3 to inhibit the JAK/STAT3, Wnt/β-catenin, Notch and PI3K/Akt pathways, respectively [117]. miR-637 is regulated by competing endogenous RNAs (ceRNAs) and is involved in five signaling pathways, including the JAK/STAT3, Wnt/β-catenin, and PI3K/Akt signaling pathways, in OC [118]. Additionally, the JAK/STAT pathway can exert effects on ovarian cancer by shaping immune cell infiltration. Interferon-mediated activation of STAT1 leads to the expression of the downstream target CXCL10, which is key to the trafficking and differentiation of effector Th1 CD4 + cells, natural killer (NK) cells and CD8 + cells [113]. Moreover, attenuation of the JAK/STAT3 signaling pathway mediated by overexpression of miR-217 can suppress M2 macrophage polarization and regulate the immune status [119].

#### Notch signaling pathway

The Notch signaling pathway is activated by the binding of ligands to Notch receptors. Following proteolytic cleavage of Notch by γ-secretase (an instrumental proteolytic enzyme in the Notch pathway), the active NICD fragment is translocated to the nucleus, where it induces the transcription of Notch target genes through interaction with CSL transcriptional regulators [120]. Aberrant Notch pathway can cause drug resistance in ovarian cancer cells, whereas Notch knockdown can increase platinum sensitivity through downregulation of ABCC1 and ABCB1 [121, 122]. In addition, inhibition of the Notch signaling pathway can induce apoptosis and reverse drug resistance. The γ-secretase inhibitor N-[N-(3,5-difluorophenacetyl)-L-alanyl]-S-phenylglycine t-butyl ester (DAPT) can induce apoptosis by downregulating Notch signaling, in turn reversing platinum resistance in ovarian cancer cells [123, 124]. In addition, suppression of Notch signaling can increase apoptosis in ovarian cancer cells in animal models and reverse resistance to cisplatin and paclitaxel [121, 125].

#### GAS6/AXL pathway

GAS6 binding to AXL leads to AXL dimerization and autophosphorylation at tyrosine residues, which results in intracellular signal transduction [126]. The GAS6/AXL pathway influences drug resistance through interactions with other signals and regulation of the tumor microenvironment (TME). For instance, AXL-related EMT mediates resistance to chemotherapy and targeted therapy [127, 128]. The GAS6/AXL pathway also confers resistance through interactions with other signaling pathways, such as the PI3K, JAK/STAT and MAPK pathways, in ovarian cancer [129]. Moreover, the role of the GAS/AXL pathway in DDR has gradually been revealed in ovarian cancer. Inhibition of AXL (via bemcentinib or MYD1-72) resensitizes ovarian cancer cells to platinum, ATR inhibitors (ATRis) and PARPis by increasing DNA damage and inducing RS [130–132]. Furthermore, GAS6/AXL signaling promotes the generation of an immunosuppressive TME by modulating the expression of MHC and PD-L1 in neoplastic cells, increasing the secretion of immunosuppressive chemokines, and interfering with the infiltration of immune cells [133]. Although miR-515-3p regulates oxaliplatin sensitivity in mucinous ovarian cancer, in part by targeting AXL [134], there is still a lack of sufficient evidence demonstrating the roles of miRNAs in regulating the GAS6/AXL pathway.

#### Transforming growth factor-beta (TGF-β) pathway

Activation of the TGF-β signaling pathway occurs via the interaction of the dimeric TGF-β ligand with its specific transmembrane receptors [135]. TGF-β signaling is transduced via downstream SMAD effectors and non-SMAD proteins, such as AKT and MAPK [136]. miRNAs can target the components of the TGF-β signaling pathway to mediate drug resistance in ovarian cancer. For instance, miR-33a-5p influences the expression of SMAD2/4 by targeting carnitine O-octanoyl transferase (CROT), which induces paclitaxel resistance in ovarian cancer [137]. Decreased miR-30a expression can result in upregulation of TGF-β and SMAD4 to ultimately activate autophagy, mediating cisplatin resistance in ovarian cancer [138]. However, miR-181a plays an unappreciated role in mediating resistance in HGSOC via the activation of TGF-β signaling by directly targeting SMAD7 [139].

The TGF-β pathway has biphasic effects and acts as a tumor suppressor at early stages but later stimulates cancer progression by impacting tumor cells and their microenvironment [135]. Aberrant activation of this pathway blocks apoptosis and confers chemoresistance on ovarian cancer cells [140]. In addition, the TGF-β pathway plays a vital role in platinum resistance via canonical downstream EMT-related molecules [141]. The TGF-β pathway also suppresses immunity within the TME and contributes to chemoresistance. Daniel Newsted et al. developed an inhibitory antibody (anti-TGFBR2) to block TGF-β signaling and showed that this antibody improved the efficacy of chemotherapy and the limited antitumor immune response [142]. Moreover, the immunosuppressive effects of the TGF-β signaling pathway can be induced via CRISPR/Cas9-mediated knockout of TGF-β receptor 2 (TGFBR2) in TILs [143].

#### MAPK pathway

RAS/RAF/MEK/ERK are the classical and key signaling mediators in the MAPK pathway, and low-grade serous carcinoma (LGSC) of the ovary and peritoneum are characterized by MAPK pathway alterations and chemoresistance [144]. Excessive activation of Ras and Erk1/2 is positively and significantly correlated with chemoresistance in ovarian cancer [145]. Both the PI3K/Akt and Ras/MAPK signaling pathways can mediate the phosphorylation of the proapoptotic protein BAD, which leads to increased platinum resistance by inhibiting apoptosis [146]. miRNAs also play regulatory roles in the MAPK pathway by interfering with its components. For example, miR-634 can directly repress GRB2, ERK2 and RSK2, hence, inhibition of the Ras-MAPK pathway restores chemosensitivity in ovarian cancer cells [147]. Low levels of miR-508/miR-18a and increased expression of MAPK1 and ERK were identified in ovarian cancer, while miR-508 mimics were found to repress MAPK1 and ERK, resulting in suppression of EMT and the malignant progression of cancer cells [148, 149].

#### Hippo/yes-associated protein (YAP) pathway

The Hippo pathway confers resistance to therapeutic agents that are commonly used to treat ovarian cancer [150, 151]. YAP and its paralog TAZ are the main downstream effectors of the Hippo–YAP pathway and act as transcriptional coactivators, and their signaling has emerged as key mechanism of drug resistance [152, 153]. YAP and TAZ mediate gene transcription by binding to TFs, such as the TEA domain family (TEAD) proteins, to promote tumor progression and resistance [153, 154]. miRNAs can regulate the expression of YAP1 and modulate the Hippo pathway, but the regulatory mechanism involved remains vague. miR-509-3p, miR-509–3-5p [155] and miR-141 [156] are associated with cisplatin resistance via YAP1 and the Hippo signaling pathway. It is hypothesized that miR-509–3-5p can directly regulate YAP1 expression by targeting its coding region [155].

### Epigenetic modifications

**Fig. 5:**
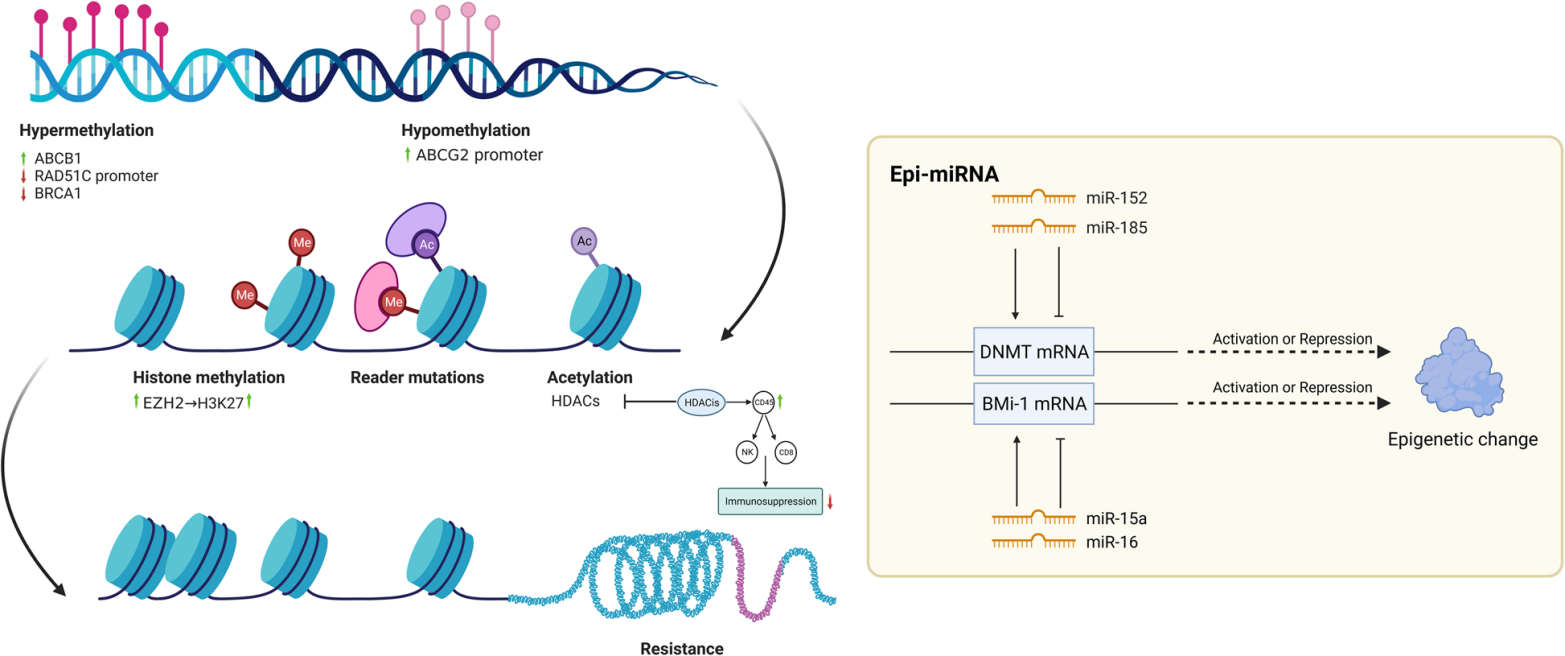
Epigenetic modification. Epigenetic processes regulate gene expression through DNA methylation, histone modification, and non-coding RNA (ncRNAs) without altered DNA sequences. Hypermethylation of ABCB1 and demethylation of ABCG2 promoter lead to chemoresistance in ovarian cancer. The loss of RAD51C promoter methylation and the downregulation of BRCA1 methylation have been verified to cause drug resistance. The specific H3K27 methyltransferase EZH2 confers chemoresistance on ovarian cancer cells through H3K27 methylation. A subclass of miRNAs, “epi-miRNAs”, can modulate epigenetic regulators to impact therapeutic responses. miR-152 and miR-185 co-contribute to the cisplatin resistance by directly targeting DNMT1, miR-15a and miR-16 directly target the Bmi-1 (a member of Polycomb complexes). They may serve as potential epigenetic therapeutic targets. Epigenetic therapy including DNMTi and HDACi can increase the number of CD45 + immune cells, active CD8 + T and NK cells in TME, reducing immunosuppression. Thus, the epigenetic therapy combined with immunotherapy may be a promising therapeutic strategy for resistant OC. (HDACs, histone deacetylases; H3K27, histone H3 lysine 27; EZH2, enhancer of zeste homolog 2; DNMTis, DNA methyltransferase inhibitors; HDACis, histone deacetylase inhibitors; Bmi-1: a member of Polycomb complexes)

Epigenetic regulation refers to the effects of heritable changes in gene expression without DNA sequence changes. DNA methylation, histone modification and noncoding RNA (ncRNA) activity (Fig. 5) are common epigenetic regulatory mechanisms [157]. Increasing evidence shows that abnormal epigenetic regulation leads to tumor drug resistance.

DNA methylation can affect therapeutic responses through various mechanisms, including affecting membrane transport, DNA repair, signaling pathway activity and apoptosis [158]. For instance, hypermethylation of ABCB1 and demethylation of the ABCG2 promoter may affect therapeutic efficacy and lead to chemoresistance in ovarian carcinoma, effects attributed to upregulation of P-gp [159, 160]. Abnormal methylation of genes involved in the PI3K-AKT, MAPK, and Wnt pathways and in EMT confers resistance on HGSOC cells [161–163]. In addition, loss of RAD51C promoter methylation and a low level of BRCA1 methylation have been verified to cause drug resistance. Homozygous RAD51C methylation and hypermethylation of BRCA1 could be predictive biomarkers for the treatment response in HGSOC [164]. Epigenetic alterations in the docking protein 2 (DOK2) gene can induce carboplatin resistance in ovarian cancer via suppression of apoptosis [165].

Histone modifications mainly include histone methylation and acetylation [166]. Min-Gyun Kim et al. confirmed the correlation between overexpression of histone deacetylases (HDACs) and cisplatin resistance in the ovarian cancer cell lines SKOV3 and OVCAR3 [167]. Recent data have provided novel insight into the role of histone H3 lysine 27 (H3K27) methylation in resistance mechanisms [168]. The specific H3K27 methyltransferase enhancer of zeste homolog 2 (EZH2) confers chemoresistance on ovarian cancer cells through H3K27 methylation [169]. In addition, Yujie Fang et al. revealed the roles of histone acetylation in a weak immune response and chemoresistance in ovarian cancer based on analysis of the TCGA and Gene Expression Omnibus (GEO) databases [170]. In terms of treatments, epigenetic therapy, including treatment with DNA methyltransferase and histone deacetylase inhibitors (DNMTis and HDACis, respectively), can increase the numbers of CD45 + immune cells, active CD8 + T cells and NK cells in the TME, reducing immunosuppression and the tumor burden through activation of type I interferon signaling in murine ovarian cancer [171, 172].

NcRNAs, comprising long ncRNAs (lncRNAs), small ncRNAs (sncRNAs) and circular RNAs (circRNAs), can regulate gene expression via epigenetic modification [173]. Most commonly, lncRNAs and circRNAs play roles in drug resistance by acting as miRNA sponges to regulate downstream gene expression [174]. “Epi-miRNAs” exert their effects by directly targeting epigenetic regulators, such as DNMTs and HDACs, or components of polycomb repressor complexes [175]. miRNAs affect mRNA transcription by binding to mRNA 3'-UTRs, leading to restoration of the expression of hypermethylated tumor suppressor genes [176]. Downregulated miR-152 and miR-185 contribute cooperatively to cisplatin resistance by directly targeting DNMT1 and may thus serve as epigenetic therapeutic targets [177]. miR-15a and miR-16 directly target the 3'-UTR of Bmi-1 (a component of Polycomb complexes), and their expression levels are significantly correlated with the Bmi-1 protein level in ovarian cancer [178].

### Other mechanisms

Indeed, determining the complex mechanisms of resistance in ovarian cancer remains highly challenging. The resistance mechanisms cross-talk with each other and may interfere by generating an immunosuppressive environment, thus resulting in drug resistance, including immunotherapy resistance. An imbalance of Treg/Th17 cells [179], M2 polarization of macrophages [180], NK-cell exhaustion [181], and aberrant expression of IFNγ [182] and PD-L1 [183, 184] mediate immunosuppression, promoting tumor progression and resistance. miRNAs, such as miR-29a-3p, miR-21-5p, miR-1246, miR-29c, and miR-424, can modulate the expression of immune-related molecules to influence the immune status. Conversely, the TME or immunotherapy can regulate the expression of many miRNAs to promote drug resistance [185, 186]. The Hedgehog (Hh) and Wnt/β-catenin pathways can also promote T-cell exclusion and checkpoint inhibitor resistance [187, 188]. However, monotherapy with the Hh pathway inhibitor vismodegib did not show any significant antitumor activity in patients with ovarian cancer in a phase II clinical trial (NCT00739661) [189]. Interestingly, although Wnt signaling is a driver of resistance in ovarian cancer, the genetic driver of Wnt signaling is largely unknown [190].

In addition to the above mechanisms, aberrations in apoptosis, ferroptosis, autophagy, and endoplasmic reticulum stress (ER stress) act simultaneously or sequentially to enable cancer cells to survive treatment with antitumor agents. miR-130a [191] and miR-142-5p [192] have been reported to modulate apoptosis by targeting XIAP. An in-depth study of ferroptosis revealed that ferroptosis played a pivotal role in acquired resistance to sorafenib [193], EGFR tyrosine kinase inhibitors [194], and immunotherapy tolerance [195]. Intriguingly, autophagic flux can be driven by paclitaxel to promote paclitaxel resistance in ovarian cancer [196] and can be regulated by miR-30a [138], miR-200c [197], and miR-133a [198]. Furthermore, as a popular research topic, ER stress has a considerable impact on drug resistance in ovarian cancer [199]. The IRE1α/XBP1s pathway activates the unfolded protein response (UPR) during ER stress, resulting in microenvironment remodeling or resistance to treatment [199, 200].

## Strategies for overcoming drug resistance

### Clinical trials targeting transmembrane transport

Overexpression of ABCB1 (also known as p-gp/MDR1) mediates increased drug efflux. Increased drug efflux makes attaining a sufficient intracellular concentration of drugs challenging, thus resulting in drug resistance [201, 202]. The ABCB1 inhibitors (verapamil and elacridar) can reverse MDR through reducing the efflux of many drugs, including paclitaxel, olaparib, doxorubicin and rucaparib [24]. Moreover, PARPi resistance was evaluated in a mouse model and was found to be reversed by coadministration of tariquidar (a P-gp inhibitor) [26]. Although preclinical studies of the response to P-gp inhibitors have been performed, clinical trials of P-gp inhibitors are limited and outdated due to the severe toxic effects of these drugs [203]. For instance, P-gp inhibition increases the intracellular accumulation of paclitaxel, leading to paclitaxel-induced peripheral neuropathy [204]. NcRNAs play key roles in the regulation of ABC transporters and their clinical implications for MDR [8]. Thus, novel strategies for post-resistance therapy include delivering ncRNA mimics or antisense oligonucleotides of ncRNAs to interfere with ncRNA-ABC transporter axes. Moreover, codelivery of miR-129-5p and doxorubicin via polypeptide nanoparticles was found to effectively overcome MDR by directly inhibiting P-gp, thereby increasing intracellular doxorubicin accumulation and enhancing chemosensitivity [205].

Recently, antibody‒drug conjugates (ADCs), which can directly deliver potent cytotoxic drugs to cancer cells with appropriate target antigens while avoiding toxic effects on healthy cells, have gained increasing attention. Currently, the only FDA-approved ADC, namely, mirvetuximab soravtansine, has attracted widespread attention in the context of ovarian cancer drug resistance. A phase III clinical trial, MIRASOL (NCT04209855), is underway to compare the efficacy of chemotherapy and mirvetuximab soravtansine in FRα-positive, platinum-resistant HGSOC. The novel ADC BA3011 can target the Axl receptor on cancer cells through conditionally active biologics technology. A phase II clinical trial is underway to evaluate the combination of BA3011 and durvalumab in patients with platinum-resistant HGSOC (NCT04918186). MUC16 is another common target for platinum-resistant ovarian cancer treatment evaluated in two completed phase I trials (NCT01335958 [206] and NCT02146313 [207]). The results showed that the anti-MUC16 ADC had a tolerable safety profile and encouraging antitumor activity in patients with platinum-resistant ovarian cancer with high MUC16 expression. Additionally, down-regulation of some miRNAs could lead to abnormal MUC16 levels in OC. Thus, their up-regulation or mimics could be potential options along with anti-MUC16 for OC patients [208]. Mesothelin is an glycoprotein overexpressed on the surface of cancer cells. Two phase I clinical trials (NCT01469793/NCT02751918) evaluated a novel anti-mesothelin ADC in platinum-resistant ovarian cancer. Conclusions drawn from these trials indicated the tolerability and promising clinical activity of anetumab ravtansine combined with PEGylated liposomal doxorubicin [209], although the results of previous trials were inconsistent. Another ADC drug Zilovertamab Vedotin, targeting ROR1, was applicated in the II-phase clinical trials (NCT04504916). ROR1 also can be targeted by miR‑382, which might serve as another option for OC [210]. Additionally, HER2, TROP2, DLL3, and Nectin-4 are major targets of ADCs. Combination strategies with ADCs have shown considerable promise as emerging therapies in further investigations and clinical trials [211].

### Clinical trials targeting DDR

The HRR pathway contributes to a key mechanism of acquired platinum and PARPi resistance in ovarian cancer. DNA repair-targeted therapy is a promising precision medicine strategy for ovarian cancer. Many clinical trials, including trials evaluating drugs targeting ATR, ATM, WEE1, checkpoint kinase 1/2 (CHK1/2), BRCA1/2 and RAD51, have been designed to evaluate interference with DDR pathways to overcome platinum and PARPi resistance in ovarian cancer.

#### ATR/ATM kinase inhibitors

ATR/ATM kinases, key molecules in DDR, are potential therapeutic targets for overcoming drug resistance in ovarian cancer. miR-203a-3p mimics and ATMis were reported to synergistically hinder OC progression, which could serve as a potential therapeutic option for OC [212]. It has been reported that ATRis can reverse PARPi resistance by blocking RAD51 loading onto DSBs and disrupting fork protection in human-derived cell lines [213]. An increasing number of clinical trials have evaluated the efficacy of ATRi or ATMi in combination with chemotherapeutic agents or PARPis. An interventional and crossover phase II randomized clinical trial (NCT02595892) was the first randomized clinical trial of an ATRi and demonstrated the benefit of adding berzosertib to gemcitabine for the treatment of platinum-resistant HGSOC [214]. M4344 enhances the activity of clinical DNA-damaging agents, including topoisomerase inhibitors, gemcitabine, cisplatin, and talazoparib, in advanced solid tumors [215]. Recently, another single-group interventional phase I trial (NCT04149145) in patients with PARPi-resistant HGSOC was just announced, in which a combination regimen of M4344 (an ATRi) plus niraparib will be evaluated.

#### WEE1 inhibitors

WEE1 is a vital target in the HRR pathway, and WEE1 inhibitors have been widely evaluated in combination with chemotherapeutic agents or PARPis in many ongoing clinical trials. A phase Ib nonrandomized, multicenter study (NCT04516447) in patients with platinum-resistant ovarian cancer evaluated the preclinical activity of ZN-c3 in combination with carboplatin, PLD, paclitaxel, and gemcitabine individually. The WEE1 inhibitor MK-1775 in combination with carboplatin or gemcitabine hydrochloride was tested in two phase II trials (NCT01164995 and NCT02272790). Adavosertib combined with chemotherapy showed preliminary therapeutic efficacy in platinum-resistant ovarian cancer, but the hematologic toxicity of this combination may limit its application [216, 217]. In addition, a phase I/II clinical trial of the WEE1 inhibitor ZN-c3 combined with niraparib was conducted in patients with platinum-resistant ovarian cancer (NCT05198804), but no results have been published. In addition, a conference abstract (ASCO 2021) reported that adavosertib alone or in combination with olaparib demonstrated efficacy in patients with PARPi resistance. Although grade 3 and 4 toxicities could be managed, they led to dose interruption and reduction (NCT03579316).

#### CHK1/2 inhibitors

Investigations of CHK1/2 inhibitors have been limited until recently. CHK1 inhibitors play a preliminary role in the clinical treatment of PARPi-resistant HGSOC by inducing DNA damage and RS [218]. miRNA‑199b‑3p suppressed CHK1 expression and EMT transition, which may represent a promising therapeutic target for ovarian cancer [219]. A phase Ia dose-escalation trial, the combination of PHI-101 (a selective CHK2 inhibitor) with a PARPi showed good safety and tolerability, and is a potential therapeutic regimen for platinum-resistant recurrent ovarian cancer [220]. In summary, the therapeutic efficacy and underlying mechanisms of CHK1/2 inhibitors are unknown, and further studies are attractive and needed.

#### Downregulation of BRCA1/2 and RAD51

The reactivation of the HRD genes BRCA1/2 and RAD51 is the genetic mechanism of PARPi resistance and confers a dismal prognosis [221]. Cediranib can potentially reverse PARPi resistance by downregulating BRCA1/2 and RAD51 and ultimately resensitizing cells to PARPis [222]. However, this combination regimen showed activity in patients with ovarian cancer who progressed on PARPi therapy in another phase II trial (EVOLVE) [221]. However, in a randomized phase II trial (BAROCCO), the combination of a PARPi and cediranib did not improve PFS in platinum-resistant ovarian cancer patients compared with chemotherapy alone [223]. The underlying mechanisms of these combination strategies have not been thoroughly elucidated.

### Clinical trials targeting signaling pathways

#### Targeting the PI3K/AKT pathway

The PI3K/AKT pathway is regarded as a common oncogenic signaling pathway. Approximately 70% of ovarian cancer patients have aberrations in the PI3K/AKT signaling pathway, and mutations in the gene encoding the catalytic subunit PIK3CA occur in 6–12% of patients [224, 225]. CYH33, a PI3Kα inhibitor, exhibited a manageable safety profile and preliminary antitumor efficacy in patients with PI3KCA-mutant ovarian cancer (NCT03544905). In addition, a phase I clinical trial (NCT04586335) is underway to further evaluate the therapeutic efficacy of CYH33 in combination with olaparib in platinum-resistant ovarian cancer. In addition, a combination regimen of PARPi and copanlisib (a PI3K inhibitor) was tested in phase I/II trials (NCT03586661 and NCT05295589) in patients with BRCA-mutated, resistant ovarian cancer. PI3K inhibition is believed to lead to downregulation of the BRCA1/2 proteins, which enhances HRR deficiency and the efficacy of PARPis. In addition, the Akt inhibitor afuresertib is under assessment in an interventional randomized clinical trial (NCT04374630) in patients with platinum-resistant ovarian, fallopian tube, or peritoneal cancer.

#### Targeting the GAS6-AXL pathway

The GAS6-AXL signaling pathway is another crucial player in drug resistance in ovarian cancer. Carboplatin/olaparib plus AVB-500, a selective inhibitor of GAS6-AXL, can increase DNA damage and RAD51 focus formation and slow replication fork progression, resulting in rapid death of ovarian cancer cells in vitro and decreased tumor burden in vivo [131]. A phase 1b trial (NCT03639246) evaluated AVB-S6-500 in combination with paclitaxel or PEGylated liposomal doxorubicin. PROC patients may derive the greatest benefit from AVB-500 treatment [226]. Another phase I/II clinical trial (NCT04019288) was designed and was commenced in 2019 to evaluate the safety and clinical benefit of durvalumab plus AVB-S6-500 (an AXL inhibitor) in platinum-resistant ovarian cancer patients. It was reported that the combination of AVB-S6-500 and durvalumab was tolerable in PROC patients [227]. Moreover, a humanized anti-AXL monoclonal antibody, tilvestamab, blocks GAS6-mediated AXL receptor activation and has been tested in platinum-resistant HGSOC patients (NCT04893551), but no results have been published.

#### Targeting the MAPK pathway

The RAS/RAF/MEK/ERK kinase pathway, also known as the MAPK pathway, participates in cancerogenesis, metastasis and resistance. Although VS-6766 (a RAF/MEK inhibitor) exhibited antitumor activity in platinum-resistant low-grade serous ovarian cancer and endometrial adenocarcinoma with RAF–RAS–MEK pathway mutations, patients later experienced progression. Thus, the use of VS-6766 in combination regimens warrants further evaluation. The combination of defactinib (a FAK inhibitor) and VS-6766 was evaluated for its pharmacodynamic activity in PROC patients (NCT03875820). In addition, combined PI3K/mTOR and ERK inhibition can reverse therapeutic resistance in ovarian cancer cell lines, but the clinical efficacy of these agents requires further preclinical determination [228]. ONC201, a dual inhibitor of Akt and ERK, is being evaluated in combination with paclitaxel for the treatment of platinum-resistant ovarian cancer in an ongoing phase II trial (NCT04055649). The unpublished results of this trial are likely to provide strong evidence for the development of novel treatment strategies.

#### Targeting the Notch pathway

The Notch pathway is linked to the proliferation, migration, and drug resistance of ovarian cancer cells [229]. Pretreatment with the γ-secretase inhibitor DAPT increased the sensitivity of PROC to platinum by downregulating the Notch pathway, suggesting a promising approach for treating patients with PROC [123, 230]. The SIERRA open-label phase Ib trial (NCT01952249) was conducted to observe the safety and efficacy of demcizumab (potent inhibitor of the Notch pathway) combined with paclitaxel for the treatment of platinum-resistant ovarian, primary peritoneal, and fallopian tube cancer. The results indicated that this combination had a manageable toxicity profile and showed a clinical benefit rate of 42% in patients with heavily pretreated platinum-resistant ovarian cancer [231].

#### Targeting the NF-κB pathway

Activation of the NF-κB pathway contributes to aggressive behaviors, mediating the oncogenic activity of DDR-related genes [232]. Furthermore, the scientific literature supports the interaction and colocalization of NF-κB and BRCA1 [233]. Denosumab, an inhibitor of RANKL (an NF-κB ligand) and NF-κB signaling, was evaluated in ovarian cancer patients with BRCA1 mutations. However, the pilot study (NCT03382574), which compared growth and metastatic spread between the denosumab and control groups, was terminated early due to the inability to enroll participants [234].

In addition, components of the cell cycle and apoptosis machineries, including topoisomerase I (NCT04029909), P53 (NCT03113487), and CDK2 (NCT05252416), could be promising treatment targets. An increasing number of early-phase clinical trials involving the glucocorticoid receptor (GR), FAK, and HER2 are underway. Although the results are pending, these studies could provide sufficient rationale for the involvement of these signaling pathways. The restoration of miR-206 expression represented a potential anti-FAK strategy to control ovarian cancer progression in EOC lines [221]. Some miRNAs were designed to target 3’-UTR of HER2 to inhibit HER2 protein expression [235]. However, the miRNA targeting drugs lacks application in clinical trials. Emerging peptide vaccines aimed to elicit a host immune response against tumor-specific antigens, such as p53, HER2, NY-ESO-1, and FRα, are being evaluated [236]. However, cancer vaccines have had limited clinical success, and research on most peptide vaccines for gynecological malignancies is still at an exploratory stage.

### Clinical trials targeting epigenetic modifications

Increased DNA methylation and histone modifications can alter the transcription of tumor suppressors and genes related to the apoptotic response to chemotherapy [224, 237]. An increasing number of trials have provided insight into the role of epigenetic modifications in the drug resistance of ovarian cancer. Researchers have attempted to overcome platinum resistance by coadministration of hypomethylating agents. For instance, guadecitabine plus carboplatin was tolerable and resulted in a detectable clinical response in patients with PROC in a phase I clinical trial [238]. However, in the phase II trial, the guadecitabine plus carboplatin group did not show any superior effect compared with the traditional chemotherapy group [239]. Furthermore, combination regimens of hypomethylating agents with PARPis or immune checkpoint inhibitors are increasingly being developed. Talazoparib and ZEN003694 (a BET inhibitor) are being evaluated in an ongoing phase II clinical trial (NCT05327010) for recurrent PARPi-resistant cancer. This series of novel therapeutic regimens has spurred the development of triplet regimens. In an ongoing phase I trial (NCT04840589), ZEN003694 and nivolumab alone or combined with ipilimumab were assessed in PROC patients. In addition, another combination therapy comprising CDX-1401 (a vaccine), atezolizumab, and guadecitabine was evaluated in a clinical trial (NCT03206047) to improve clinical efficacy. These innovative clinical trials are anticipated to provide therapeutic opportunities for drug-resistant patients.

## Conclusion

With the increasing use of novel therapeutic drugs for ovarian cancer, the development of later-line treatments has been under enormous pressure. Recently, resistance to a variety of therapeutic drugs, such as PARPis, angiogenesis inhibitors, and immune checkpoint inhibitors, has been found to occur. In the past, therapeutic agents for ovarian cancer have been limited, and researchers have usually described the underlying mechanism and explored therapeutic strategies for overcoming resistance based on the drug classification. However, as increasing numbers of new agents are applied in clinical practice, the resistance mechanisms of these various new drugs must be identified, and these mechanisms may be similar or even identical to those of other drugs. Thus, the classification of drug resistance should not be confined to the drug category, and we should attempt to obtain insight into classification of resistance based on molecular mechanisms. The concept of drug resistance classification provides a sound basis for further research to develop more precise reversal strategies.

Although the resistance mechanisms of different agents are complicated, we classified miRNA-mediated mechanisms into four categories: abnormalities in transmembrane transport, dysregulation of DDR, dysregulation of signaling pathways and epigenetic modification. On the basis of the above four mechanisms, clinical trials of new agents are underway to overcome drug resistance. Notably, ADCs, a current research hotspot, hold promise for overcoming resistance in patients with ovarian cancer. The FDA's approval of mirvetuximab soravtansine-gynx for FRα-positive, platinum-resistant HGSOC was based on Study 0417 (SORAYA, NCT04296890) [240]. Thus, many additional ADCs against various targets, including NaPi2b, HER2/3, mesothelin, and MUC16, which are expressed in ovarian cancer, are under investigation [241]. Future innovative studies and targeted therapies with ADCs will provide opportunities for reversing drug resistance in ovarian cancer. In addition, another potential approach for reversing resistance is based on miRNAs [242]. Codelivery of miRNAs with chemotherapeutic agents is a promising option for overcoming resistance, but further investigations of the underlying mechanism and the clinical application of this strategy are needed [243]. Polypeptide nanoparticles carrying doxorubicin and miR-129-5p could be a promising and synergistic strategy to overcome drug resistance in ovarian cancer [205].

In the context of the increasing number of novel agents, our summary of the four resistance mechanisms of ovarian cancer provides a new concept for resistance classification by molecular mechanism, not by drug category. Given the intersections between drug resistance mechanisms, this concept is likely to result in the realization of “two birds with one stone” effects on the reversal of drug resistance in ovarian cancer. Furthermore, these findings are anticipated to have broad implications for the development of precise therapeutic approaches for reversing drug resistance in ovarian cancer. On this basis, umbrella trials can be carried out to explore the diagnostic and therapeutic targets of the four resistance mechanisms, and this may be a direction of future researches on drug resistance in ovarian cancer.

### Supplementary Information

**Additional file 1: Figure S1/S2.** The summary of flow charts of clinical trials in I/II phase. The components in these flow chart include inclusion criteria, sample size, study duration, study arms, and study endpoints of these I-phase clinical trials about resistant ovarian cancer.

## Data Availability

No datasets were generated or analysed during the current study.

## Abbreviations

ADC: Antibody–drug conjugate
ATRi: Ataxia telangiectasia and Rad3-related protein inhibitor
ATMi: Ataxia telangiectasia mutated protein inhibitor
BCRP: Breast cancer drug resistance protein
BER: Base excision repair
ceRNA: Competitive endogenous RNA
CHK1/2: Checkpoint kinase 1/2
CSC: Cancer stem cell
DAPT: N-[N-(3,5-Difluorophenacetyl)-L-alanyl]-S-phenylglycine t-butyl ester
DDR: DNA damage repair
DHMEQ: Dehydroxymethylepoxyquinomicin
DNMTi: DNA methyltransferase inhibitor
DOK2: Docking protein 2
DYNLL1: Dynein light chain 1
EAH2: Zeste homologue 2
EMT: Epithelial mesenchymal transformation
ER: Endoplasmic Reticulum
FANC: Fanconi anemia complementation group
GST: Glutathione transferase
HDACs: Histone deacetylase
Hh: Hedgehog
HRD: Homologous recombination deficiency
HRR: Homologous recombination repair
HGSOC: High-grade serous ovarian cancer
HSP: Heat shock protein
LGSC: Low grade serous carcinoma
LRP: Lung drug resistance-related protein
MMR: Mismatch repair
MRP: Multidrug resistance-related protein
NCCN: National Comprehensive Cancer Network
ncRNA: Non-coding RNA
NHEJ: Non-homologous end junction
NER: Nucleotide excision repair
OS: Overall survival
PARPi: Poly ADP-ribose polymerase inhibitor
PFS: Progression-free survival
P-gp: P-glycoproteins
SHLD: Shieldin
TGF-β: Transforming growth factor-beta
TIL: Tumor-infiltrating lymphocytes
TLS: Translesion DNA synthesis
TEAD: TEA domain family
UCA1: Urothelial carcinoma-associated 1
3’-UTR: 3’-Untranslated region
XIAP: X-linked inhibitor of apoptosis
YAP: Yes-associated protein
