# Supplementary figures and images for: Drug resistance in ovarian cancer: from mechanism to clinical trial
Source: Mol Cancer. 2024 Mar 28;23:66. doi: 10.1186/s12943-024-01967-3 (PMC10976737; doi:10.1186/s12943-024-01967-3)

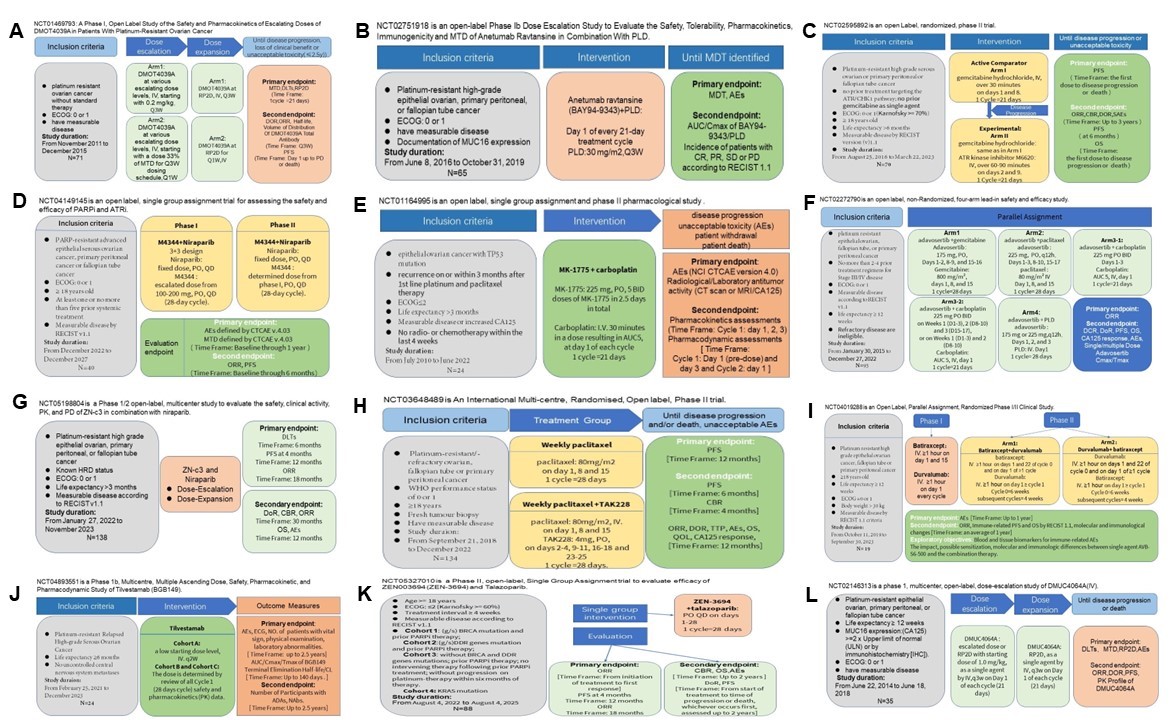

Supplement: Supplementary file 1 — Additional file 1: Figure S1/S2. The summary of flow charts of clinical trials in I/II phase. The components in these flow chart include inclusion criteria, sample size, study duration, study arms, and study endpoints of these I-phase clinical trials about resistant ovarian cancer. [file 12943_2024_1967_MOESM1_ESM.zip › 12943_2024_1967_MOESM1_ESM.jpg]

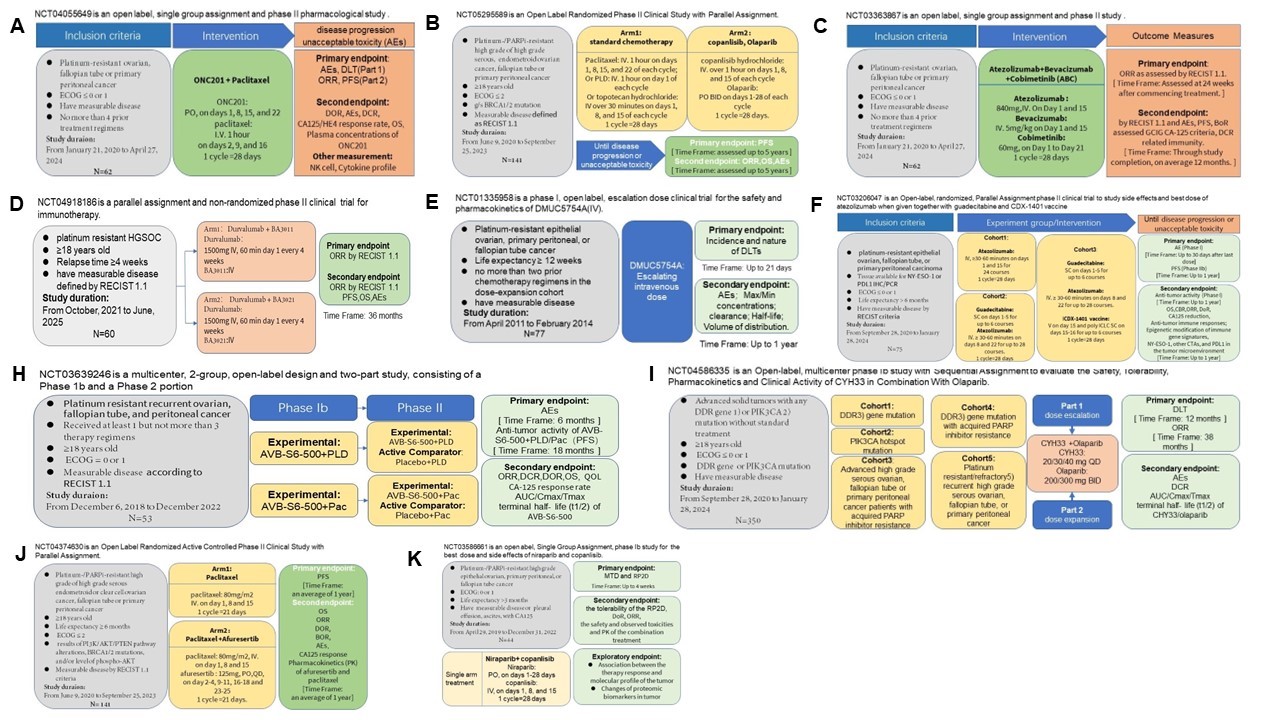

Supplement: Supplementary file 1 — Additional file 1: Figure S1/S2. The summary of flow charts of clinical trials in I/II phase. The components in these flow chart include inclusion criteria, sample size, study duration, study arms, and study endpoints of these I-phase clinical trials about resistant ovarian cancer. [file 12943_2024_1967_MOESM1_ESM.zip › 12943_2024_1967_MOESM2_ESM.jpg]
